# A simulation study of the cost of cerebral vasospasm treatments with clazosentan: A mathematical model using time-driven activity-based costing

**DOI:** 10.1371/journal.pone.0340076

**Published:** 2026-01-02

**Authors:** Jieyu Zhao, Kota Kurisu, Kazuki Ohashi, Toshiya Osanai, Katsuhiko Ogasawara, Miki Fujimura

**Affiliations:** 1 Faculty of Health Sciences, Hokkaido University, Sapporo, Hokkaido, Japan; 2 Faculty of Medicine and Graduate School of Medicine, Hokkaido University, Sapporo, Hokkaido, Japan; 3 Division of Advanced Stroke Therapy and Health Economics, Graduate School of Medicine, Hokkaido University, Sapporo, Hokkaido, Japan; 4 Graduate School of Engineering, Muroran Institute of Technology, Muroran, Hokkaido, Japan; Instituto Butantan, BRAZIL

## Abstract

In this work, we aimed to assess the impact of clazosentan on clinical labour time costs within Japan’s value-based healthcare system using time-driven activity-based costing. Time-driven activity-based costing was employed to analyse the labour time costs associated with preventing cerebral vasospasm following aneurysmal subarachnoid haemorrhage. Time-driven activity-based costing simplifies cost analysis by utilising time as the primary cost driver. We compared two treatment approaches: conventional therapy with fasudil hydrochloride and postoperative therapy with clazosentan. Scenario and sensitivity analyses were performed to assess the impact of physicians’ costs on the results. The use of clazosentan for the prevention of cerebral vasospasm significantly reduced human resource costs, particularly in cases where symptomatic vasospasm did not occur, yielding savings of approximately 51,343 yen. The greatest cost reductions were observed among nursing staff, with a 30% decrease in the absence of symptomatic vasospasm and a 15% reduction when symptomatic vasospasm was present. The cost reductions for physicians were comparatively smaller, particularly in cases where symptomatic vasospasm occurred. Sensitivity analyses indicated that clazosentan reduced overall costs by approximately 35,000–50,000 yen; however, costs increased in the presence of symptomatic vasospasm. Clazosentan for subarachnoid haemorrhage treatment significantly reduces human resource costs, especially in nursing staff. These findings support the potential of clazosentan for broader clinical use, given its cost-savings and clinical benefits in reducing cerebral vasospasm following aneurysmal subarachnoid haemorrhage.

## Introduction

Stroke is a leading cause of death and disability worldwide [1]. Subarachnoid haemorrhage (SAH), primarily caused by the rupture of cerebral aneurysms, often results in severe neurological impairment or death. Despite extensive research efforts aimed at improving outcomes in aneurysmal SAH (aSAH), a recent nationwide study [2] reported that improvements in haemorrhagic stroke outcomes lag behind those observed in ischaemic stroke. Among post-SAH complications, delayed cerebral ischaemia—mainly resulting from cerebral vasospasm—is a critical therapeutic target.

Clazosentan, an endothelin-1 receptor antagonist, has shown promise as a prophylactic agent against vasospasm. Large-scale clinical trials [3,4] have demonstrated that clazosentan significantly reduces the incidence of symptomatic vasospasm (sVSP) and improves clinical outcomes in patients with aSAH, achieving more than a 50% reduction in vasospasm-related morbidity and all-cause mortality. Moreover, patients treated with clazosentan experience a significantly lower rate of vasospasm-related symptomatic infarction compared to those treated with fasudil, a Rho kinase inhibitor [5].

In 2023, the Center for Outcomes Research and Economic Evaluation for Health reported that the incremental cost-effectiveness ratio of clazosentan ranged from 2,000,000–5,000,000 yen per quality-adjusted life year [6], suggesting that clazosentan is generally considered cost-effective within the Japanese healthcare context. However, clazosentan and other sVSP preventive treatments require varying levels of personnel, equipment, and material resources, depending on their complexity. Time-driven activity-based costing (TDABC) is a method that can quantify this complexity by measuring treatment costs based on actual time spent on care delivery.

Japan’s healthcare reimbursement system operates under a value-based payment model. Porter [7] defines value in healthcare as the outcomes achieved relative to the costs incurred to achieve them. Therefore, the accurate understanding, monitoring, and evaluation of healthcare expenditures are essential [8,9]. Bottom-up micro-costing, which identifies patient-specific resource utilisation and hospital-specific unit costs, is frequently used to assess institutional healthcare expenditures. In this context, TDABC provides a powerful tool to capture patient-level resource consumption by quantifying actual clinical time allocated to each patient [7].

This study, conducted from the perspective of hospital administrators, focused on evaluating resource utilisation costs to assess the economic viability of clazosentan. We specifically calculated human resource costs and compared these across different categories of healthcare professionals treating patients with SAH with and without the use of clazosentan at our institution.

## Materials and methods

### Analysis methods and materials

This study was based entirely on a simulation using a TDABC model, and no individual patient data or biological samples were used. TDABC, introduced by Kaplan and Anderson at Harvard Business School in 2007, was developed to enhance the traditional activity-based costing approach [10–12]. TDABC simplifies and accelerates costing by converting all cost drivers into a single cost driver—time—without compromising the accuracy of the data [10,13]. When appropriately designed and implemented, TDABC facilitates the identification of opportunities to streamline patient care by aligning resource utilisation with actual patient needs [14,15].

In clinical practice, postoperative prophylactic treatment for sVSP typically includes a conventional regimen involving intravenous administration of fasudil hydrochloride. However, to reflect the clinica trial in Japan [4], this study assumes that the control group does not receive treatment with fasudil hydrochloride.In contrast, the ‘with clazosentan’ group was modeled as those who hypothetically received postoperative clazosentan as a prophylactic measure.

To address procedural uncertainty and variability in individual costs, a probabilistic sensitivity analysis was conducted using the Monte Carlo method. To estimate the treatment costs of patients with aSAH, the TDABC method was employed as follows: (1) process mapping was undertaken to define each step in the aSAH care pathway, including time estimates for each activity, and (2) personnel capacity cost rates were calculated to compare diagnostic and surgical approaches in terms of cost. A simplified model pathway was developed based on established clinical practice guidelines. The computed labour costs were subsequently applied to each surgical modality—coiling and clipping—for comparative purposes. Labour cost data for healthcare professionals, including physicians, nurses, and ancillary staff, were obtained from the Basic Survey on Wage Structure (Table 1) [16].

**Table 1:** Human resource costs referenced from the basic survey on wage structure.

Age range (Years)	Doctor (%)	Nurse (%)	Radiographer (%)
**mean**	5924.9	2,143.9	2,143.6
**20–24**	―	1,767.1 (12.7)	1,570.5 (7.4)
**25–29**	*^1^2,775.7 (16.6)	2,009.6 (14.7)	1,715.5 (18.7)
**30–34**	4,010.4 (17.3)	2,047.9 (10.6)	1,764.2 (10.8)
**35–39**	5,777.0 (13.3)	2,097.0 (9.9)	2,077.8 (11.6)
**40–44**	*^2^ 6,141.2 (11.9)	2,214.7 (12.7)	2,359.1 (10.9)
**45–49**	8,247.3 (9.2)	2,332.1 (14.2)	2,408.8 (14.0)
**50–54**	7,561.5 (13.0)	2,326.1 (11.7)	2,658.4 (12.4)
**55–59**	*^3^8,351.2 (11.9)	2,370.9 (9.4)	2,737.9 (10.1)
**60–64**	8,004.1 (6.8)	2,110.4 (4.1)	2,096.4 (4.1)

*^1^ Resident doctor, *^2^ Specialist doctor, *^3^ Trainee doctor.

For analysis, patients undergoing neurosurgical procedures were stratified into two primary groups: those who developed cerebral vasospasm and those who did not. Each of these was further subdivided based on whether clazosentan was used, resulting in four subgroups (Table 2). Economic comparisons between these subgroups were conducted in pairs to assess the cost-effectiveness of clazosentan.

**Table 2:** Comparison of clazosentan use between patients with and without cerebral vasospasm.

Group	Subgroup
**Without symptomatic vasospasm: sVSP(-)**	Not using clazosentan: Claz(-)
	Using clazosentan: Claz(+)
**With symptomatic vasospasm: sVSP(+)**	Not using clazosentan: Claz(-)
	Using clazosentan: Claz(+)

Claz, clazosentan; sVSP, symptomatic vasospasm.

### Process maps and time estimates

**Fig 1:**
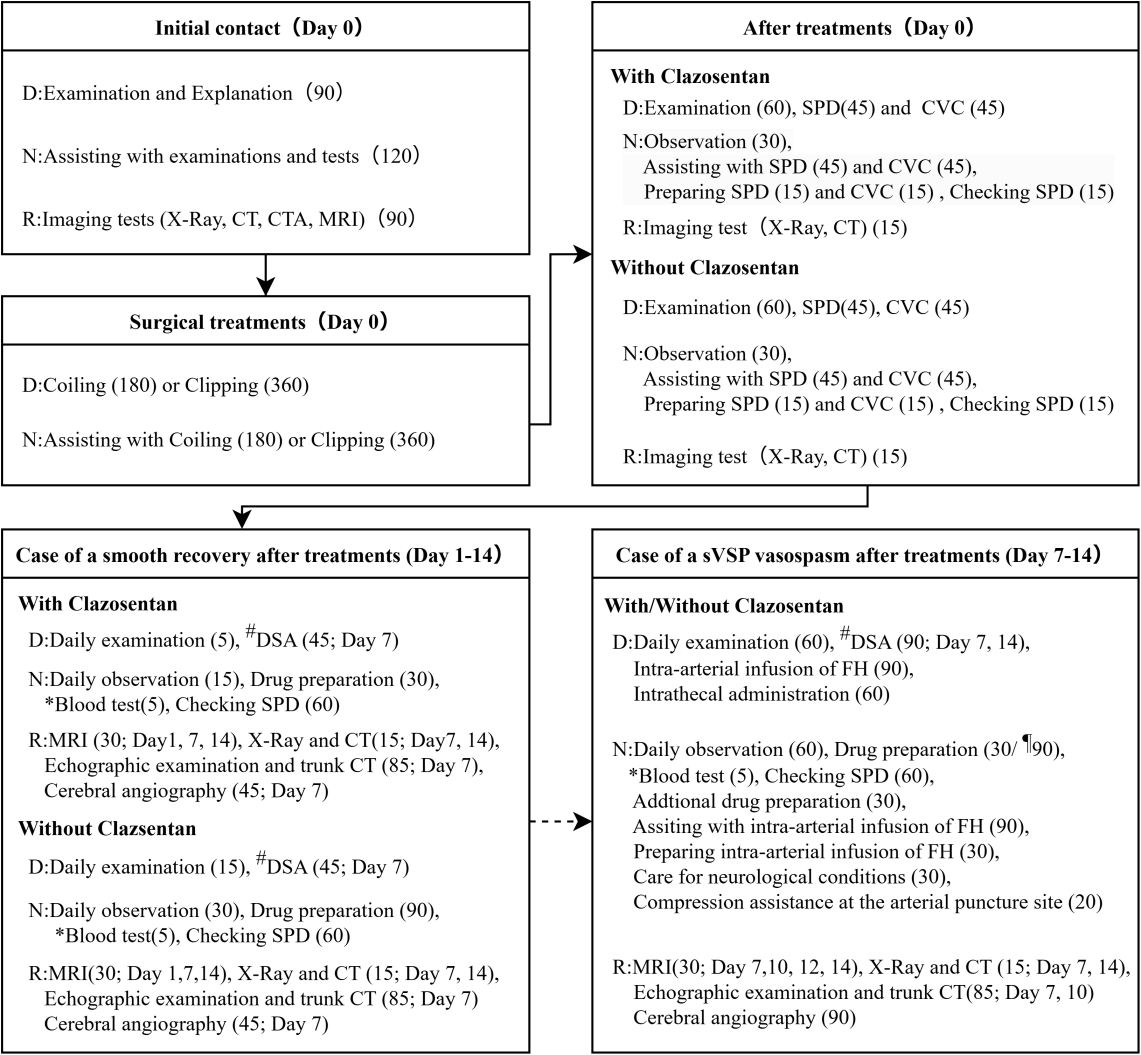
Treatment process for acute subarachnoid haemorrhage. Values in parentheses indicate the time required for each procedure (minutes). D, doctor; N, nurse; R, radiographer; CT, computed tomography; CTA, computed tomography angiography; MRI, magnetic resonance imaging; SPD, spinal drainage; CVC, central venous catheter; DSA, digital subtraction angiography; FH, fasudil hydrochloride. *#*, coiling; *¶*, without clazosentan.

A previous clinical trial demonstrated that clazosentan significantly reduced the incidence of vasospasm-related morbidity and all-cause mortality following aneurysm coiling (from 28.8% to 13.6%; relative risk reduction: 53%; 95% confidence interval [CI]: 17%–73%) and clipping (from 39.6% to 16.2%; relative risk reduction: 59%; 95% CI: 33%–75%) [4]. In this study, the acute phase of SAH was defined as the period of approximately 14 days from hospital admission to discharge. This timeframe encompassed diagnostic procedures, surgical interventions, and daily care activities (Fig 1). Coiling and clipping were employed as the primary surgical treatments during this acute phase. Subsequently, four care pathways were subjected to economic analysis based on the type of surgical procedure (coiling or clipping) and whether clazosentan was administered.

A care process model was developed using clinical expertise and institutional protocols. Notably, even with prophylactic medication, the occurrence of aSAH cannot be fully prevented and may vary in severity and duration of treatment. The treatment duration in affected patients was found to vary considerably. A decision-tree model was constructed based on data from a randomised clinical trial of clazosentan [4]. This trial employed an intention-to-treat analysis, which included patients in whom clazosentan administration was discontinued, and excluded SAH cases classified as World Federation of Neurosurgical Societies grade 5.

**Fig 2:**
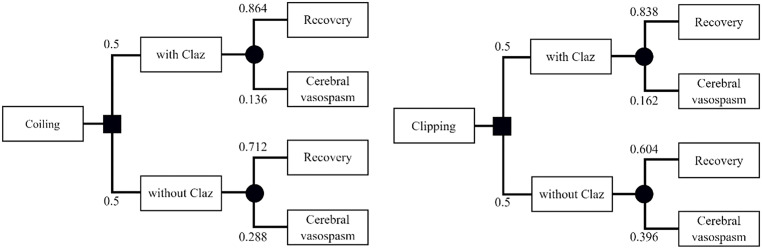
Decision tree model for the treatment of aneurysmal subarachnoid haemorrhage. Half of the patients received clazosentan following coiling or clipping and subsequently transitioned to either recovery or symptomatic cerebral vasospasm (sVSP), based on the probabilities indicated at each node (black circles). Claz, clazosentan.

According to the trial results, the incidence of cerebral vasospasm in patients who underwent coiling with and without clazosentan was 13.6% and 28.8%, respectively. In patients who underwent clipping, the corresponding rates were 16.2% and 39.6%, respectively (Fig 2) [4].

The time and human resources required for each step in the care pathway were estimated based on the expert opinions of clinicians experienced in SAH management (Table 3). A triangular distribution was applied to account for uncertainty and variability in clinical cases [17]. For procedures where detailed data were unavailable, the minimum and maximum values were set as half and twice the most likely value (mode), respectively.

**Table 3:** Parameters in the care process.

Detail	Distribution mode (min, max)	Profession	Personnel requirements	Schedule
Initial contact				
Examination and Explanation	90 (45, 180)	Physician	1	Day 0
Assisting with examination and tests	120 (60, 240)	Nurse	1	
Imaging tests (X-ray, CT, CTA, and MRI)	90 (45, 180)	Radiology technician		
Coiling	180 (120, 360)	Physician	3	
Assisting with coiling		Nurse	2	
Clipping	360 (180, 600)	Physician	3	
Assisting with clipping		Nurse	2	
After treatments				
Examination	60 (30, 120)	Physician	1	Day 0
Observation	30 (15, 60)	Nurse	1	
Imaging test (X-ray and CT)	15 (7.5, 30)	Radiology technician	1	
Insertion of spinal drainage	45 (15, 60)	Physician	1	
Assisting with insertion of spinal drainage		Nurse	1	
Preparing insertion of spinal drainage	15 (7.5, 30)	Nurse	1	
Checking spinal drainage	15 (7.5, 30)	Nurse	1	
Insertion of central venous catheter	45 (15, 60)	Physician	1	
Assisting with insertion of central venous catheter		Nurse	1	
Preparing of insertion of central venous catheter	15 (7.5, 30)	Nurse	1	
Case of a smooth recovery after treatments				
Daily examination	5 (2.5, 10)	Physician	1	Days 1–14
Without clazosentan	15 (7.5, 30)		1	
^#^DSA	45 (30, 120)	Physician	1	Day 7
Daily observation	15 (7.5, 30)	Nurse	1	Days 1–14
Without clazosentan	30 (15, 60)		1	
Drug preparation	30 (15, 60)	Nurse	1	
Blood test	5 (2.5, 10)	Nurse	1	7 days
Checking spinal drainage	60 (30, 90)	Nurse	1	Days 1–14
MRI	30 (15, 60)	Radiology technician	1	Days 1, 7, 14
X-ray and CT	15 (7.5, 30)	Radiology technician	1	Days 7, 14
Echographic examination and trunk CT	85 (42.5, 170)	Radiology technician	1	Day 7
Cerebral angiography	45 (45, 100)	Radiology technician	1	
Case of a cerebral vasospasm after treatments (Days 7–14)				
Daily examination	60 (30, 120)	Physician	1	Days 7–14
^#^DSA	90 (45, 100)	Physician	2	Days 7, 14
Intra-arterial infusion of FH	90 (45, 100)	Physician	2	Days 7–14
Assisting with intra-arterial infusion FH		Nurse	2	
Preparing intra-arterial infusion of FH	30 (15, 45)	Nurse	2	
Intrathecal administration	60 (15, 100)	Physician	1	Days 7–14
Daily observation	60 (15, 90)	Nurse	1	Days 7–14
Drug preparation	30 (15, 60)	Nurse	1	Days 7–14
Without clazosentan	90 (45, 135)	Nurse	1	Days 7–14
Additional drug preparation	30 (15, 60)	Nurse	1	Days 7–14
Checking spinal drainage	60 (30, 90)	Nurse	1	Days 7–14
Care for neurological conditions	30 (5, 60)	Nurse	1	Days 7–14
Compression assistance at the arterial puncture site	20 (5, 30)	Nurse	1	Days 7–14
MRI	30 (15, 60)	Radiology technician	1	Days 7, 10, 12, 14
X-ray and CT	15 (7.5, 30)	Radiology technician	1	Days 7, 14
Echographic examination and trunk CT	85 (42.5, 170)	Radiology technician	1	Days 7, 10
Cerebral angiography	90 (45, 100)	Radiology technician	1	Days 7–14

CT, computed tomography; CTA, computed tomography angiography; DSA, digital subtraction angiography; FH, fasudil hydrochloride; MRI, magnetic resonance imaging.

### Scenario and sensitivity analysis

Three scenario analyses were conducted to assess the impact of variations in physician costs. In each scenario, the primary physician was assumed to be a resident, specialist, or trainer-level physician, with corresponding hourly costs of 2,775.7, 6,141.2, and 8,351.2 yen/h, respectively. A probabilistic sensitivity analysis was performed using Monte Carlo simulations to evaluate the impact of uncertainty in model parameters—such as care duration and human resource costs—on the results. Procedure times were sampled using a triangular distribution, while hourly costs for healthcare workers were sampled from the distribution reported in a national wage survey [18].

The care process was evaluated through 10,000 iterations, while the decision-tree model was assessed using 1,000 iterations, each simulating 10,000 individuals. All analyses were conducted using R version 4.3.2 (R Foundation for Statistical Computing, Vienna, Austria) and RStudio version 2024.04.1 + 748, which facilitated the handling of variability in input parameters.

### Ethical statement

This study evaluates a cost through simulations using a mathematical model and does not involve any direct data or samples from subjects; therefore, ethical review is deemed unnecessary.

## Results

**Fig 3:**
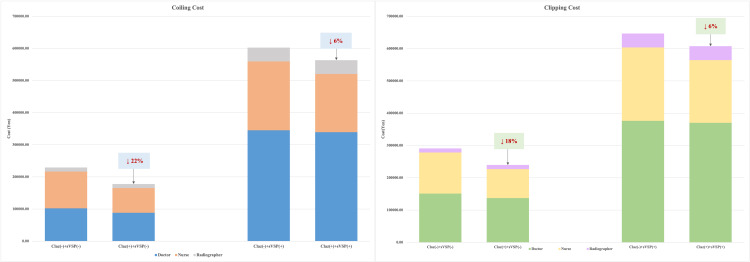
Total cost differences for patients treated with and without clazosentan across two treatment modalities: Coiling (left) and clipping (right).

The use of clazosentan to prevent cerebral vasospasm, resulted in time and cost savings for healthcare workers, irrespective of whether coiling or clipping was employed (Table 4). When sVSP did not occur, treatment costs were 51,342.98 yen lower in the clazosentan group compared to the control. In cases where sVSP occurred, the cost savings amounted to 39,155.35 yen. As shown in Fig 3, among patients who did not develop sVSP and underwent coiling or clipping, clazosentan reduced human resource costs by 22% and 18%, respectively. In contrast, among patients who experienced sVSP, the reduction in labour costs was minimal—approximately 6% for both surgical approaches.

**Table 4:** Total cost and difference for groups with and without clazosentan for patients with and without spasms under different treatments.

		Coiling		Clipping	
		Total	Difference	Total	Difference
**sVSP (-)**	Claz(-)	229,228.38	51,342.98	290,971.93	51,342.98
	Claz(+)	177,885.40		239,628.95	
**sVSP (+)**	Claz(-)	602,564.35	39,155.35	646,533.30	39,155.35
	Claz(+)	563,409.00		607,377.95	

Claz, clazosentan; sVSP, symptomatic vasospasm.

**Fig 4:**
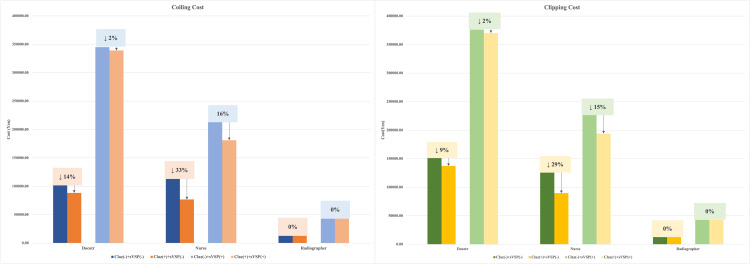
Changes in labour costs for doctors, nurses, and radiographers in groups with and without clazosentan. Changes are shown separately for coiling (left) and clipping (right).

The effect of clazosentan on labour costs varied across healthcare professions. The reduction in nursing costs was greater in non-vasospasm cases than in vasospasm cases, consistent with Table 5. Clazosentan reduced nursing costs by approximately 30% when sVSP did not occur, and by at least 15% when it did (Fig 4). Conversely, the reduction in physician labour costs was more modest. In the presence of sVSP, clazosentan reduced physician labour costs by only 2%. When sVSP was not observed, physician labour costs were reduced by 9% and 14% for clipping and coiling, respectively. No change was observed in the labour costs for radiologists.

**Table 5:** Cost differences among doctors, nurses, and radiographers in groups with and without clazosentan.

Difference				
		Coiling and Clipping		
		Doctor	Nurse	Radiographer
**sVSP (-)**	Claz(-)	13,824.68	37,518.29	0.00
	Claz(+)			
**sVSP (+)**	Claz(-)	5,924.86	33,230.49	0.00
	Claz(+)			

Claz, clazosentan; sVSP, symptomatic vasospasm.

When labour costs were stratified by physician seniority, clazosentan yielded the highest savings for trainer-level physicians in cases without sVSP, amounting to 19,486.08 yen (Table 6), approximately three times greater than the savings for resident physicians. In contrast, when sVSP occurred, cost savings were more modest, at 2,775.74, 6,141.21, and 8,351.18 yen for residents, specialists, and trainer physicians, respectively.

**Table 6:** Cost differences for the three experience levels of doctors in groups with and without clazosentan.

Difference				
		Coiling and Clipping		
		Resident doctor	Specialist doctor	Trainer doctor
**sVSP (-)**	Claz(-)	6,476.73	14,329.49	19,486.08
	Claz(+)			
**sVSP (+)**	Claz(-)	2,775.74	6,141.21	8,351.18
	Claz(+)			

Claz, clazosentan; sVSP, symptomatic vasospasm.

**Fig 5:**
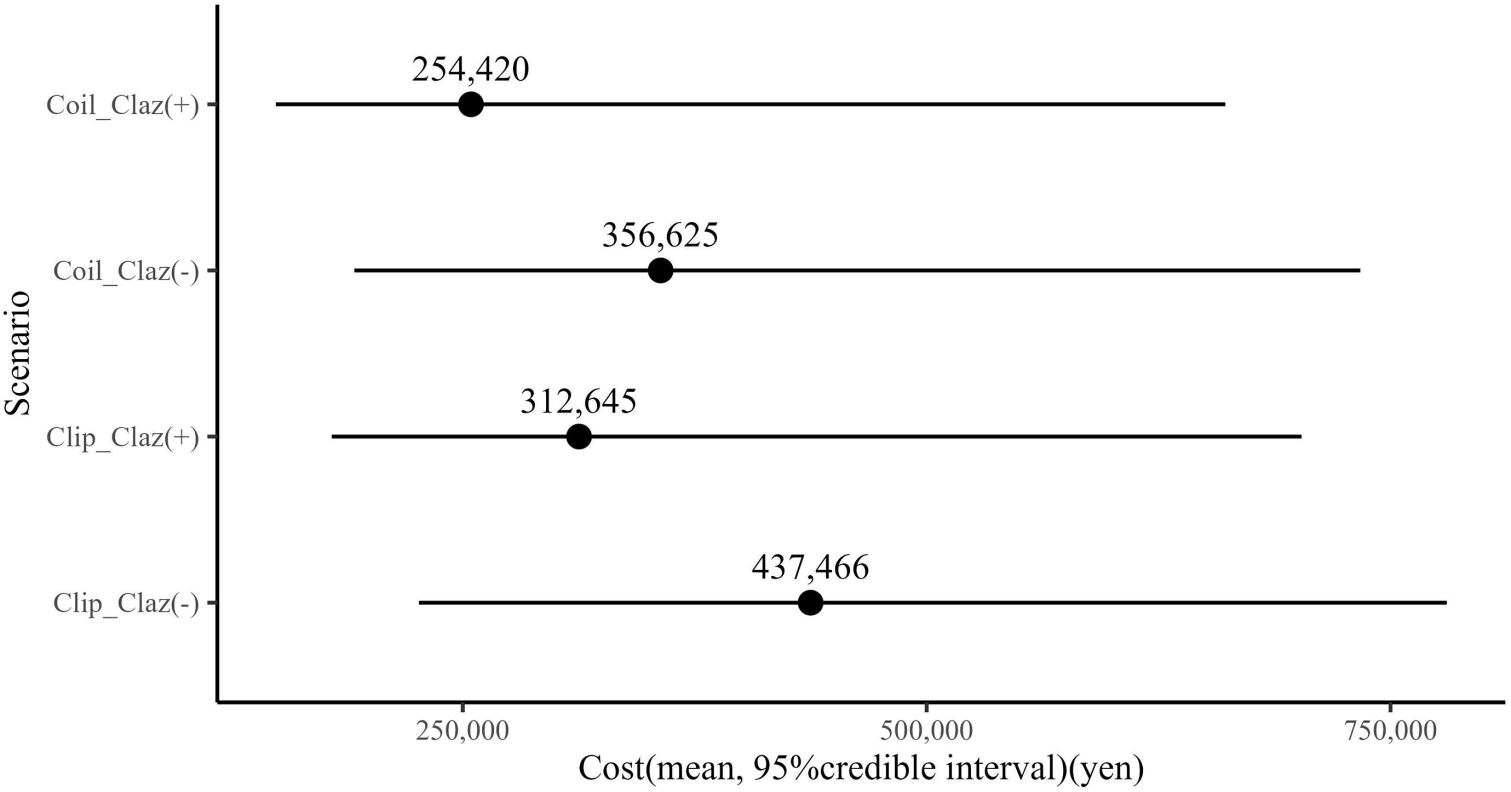
Impact of clazosentan effectiveness on total cost. Each data point represents results for 10,000 hypothetical patients undergoing each treatment pathway as modelled in the decision tree. This process was repeated with 1,000 iterations to estimate the mean and 95% credible intervals. Claz, clazosentan; sVSP, symptomatic vasospasm; SD, standard deviation.

**Fig 6:**
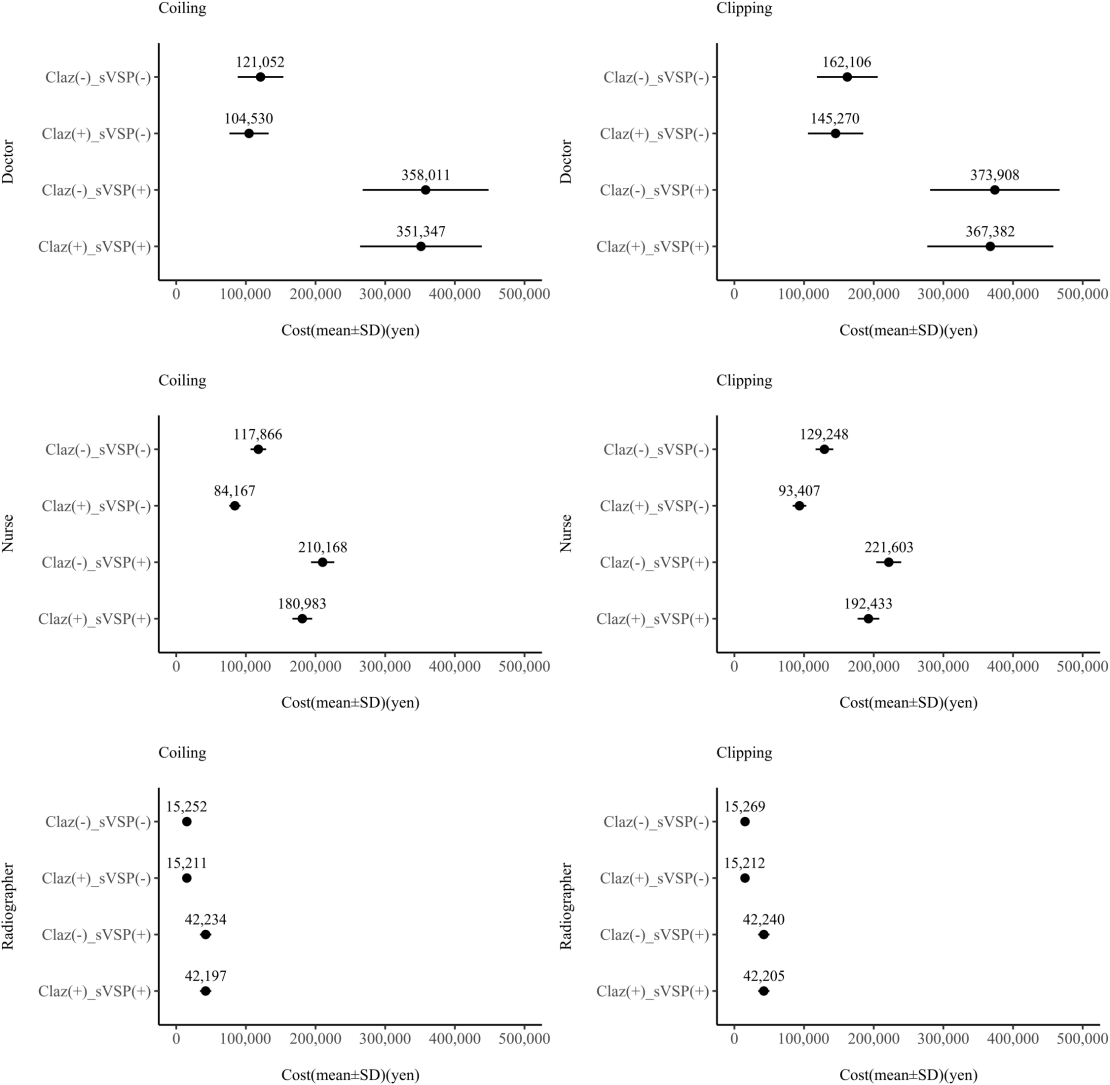
Distribution of labour costs by professional role. Left: coiling results. Right: clipping results. Top row: doctors; middle row: nurses; bottom row: radiographers. Claz, clazosentan; sVSP, symptomatic vasospasm; SD, standard deviation.

**Fig 7:**
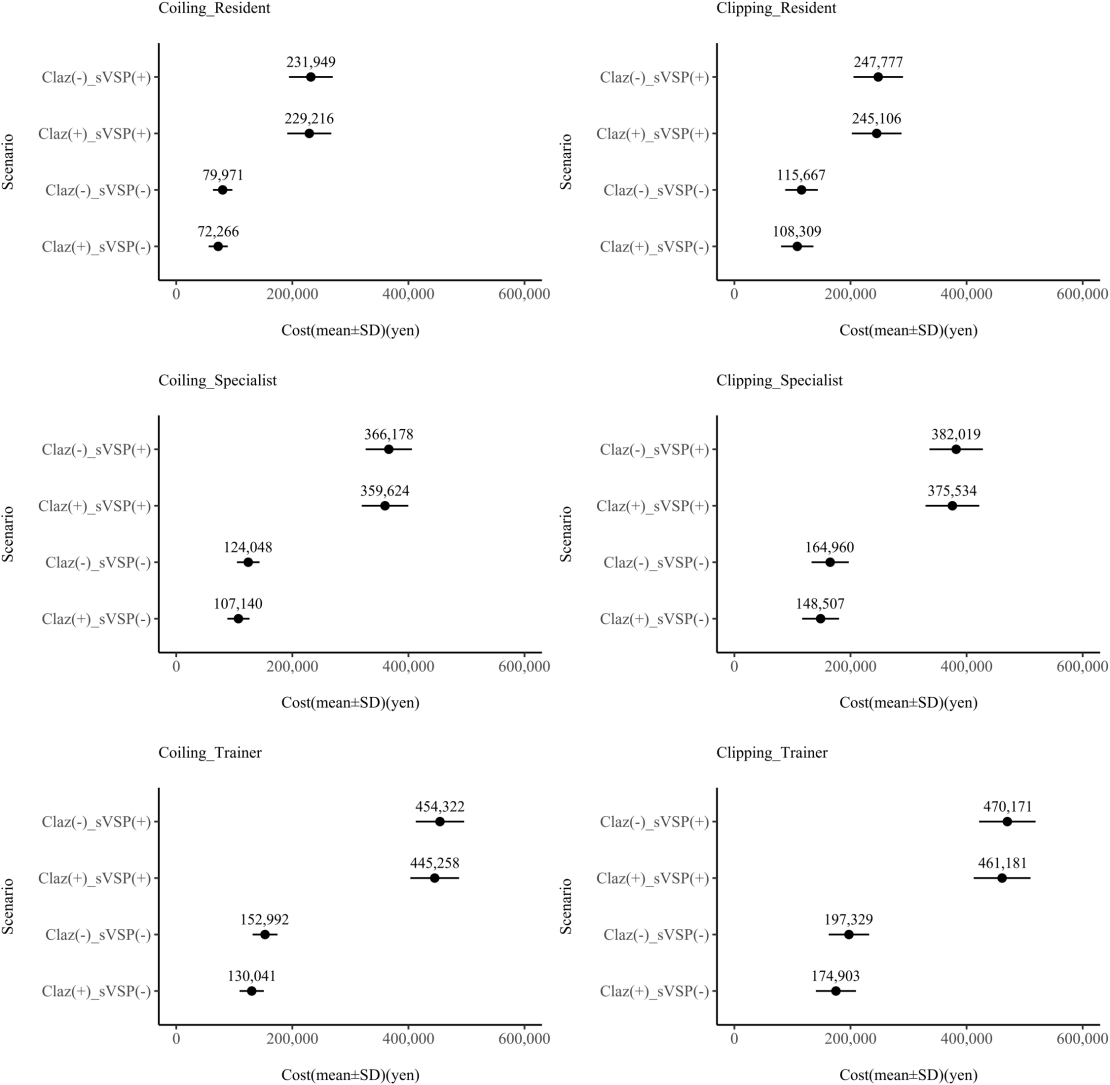
Distribution of labour costs by physician experience level. Left: coiling results. Right: clipping results. Top row: residents; middle row: specialists; bottom row: trainers. Claz, clazosentan; sVSP, symptomatic vasospasm.

The sensitivity analysis, accounting for uncertainties in procedure time and labour costs, demonstrated a reduction in human resource costs by approximately 35,000–50,000 yen when clazosentan was administered with coiling and clipping (Table 7). In addition, sensitivity analysis using a decision tree model was conducted to evaluate the impact of clazosentan’s effectiveness. According to the result with added differences in the incidence of spasms, the administration of clazosentan reduced labour costs by 100,000 and 120,000 yen for coiling and clipping, respectively (Fig 5). Regarding professional differences, clazosentan use contributed to nurses’ costs (Fig 6). The labour costs for physicians associated with coiling and clipping procedures appear to depend on their level of experience, with this trend consistently observed for both coiling and clipping procedures (Fig 7).

**Table 7:** Sensitivity analysis of procedure time and labour costs.

	Coiling			Clipping		
	Mean	SD	2.5^th^ percentile–97.5^th^ percentile	Mean	SD	2.5^th^ percentile–97.5^th^ percentile
**Claz(–), sVSP(–)**	254,170	36,333	187,554–325,882	306,623	48,852	218,346–407,411
**Claz(+), sVSP(–)**	203,908	31,280	148,406–267,472	253,889	44,674	175,834–346,064
**Claz(–), sVSP(+)**	610,413	92,856	434,034–768,860	637,751	96,610	455,746–810,815
**Claz(+), sVSP(+)**	574,526	89,791	404,347–728,144	602,020	94158	423,849–771,391

Claz, clazosentan; sVSP, symptomatic vasospasm; SD, standard deviation.

## Discussion

This study demonstrates that the use of clazosentan to prevent sVSP significantly reduces labour costs for healthcare workers involved in both coiling and clipping procedures. Specifically, clazosentan use is associated with cost savings of 51,342.98 and 39,155.35 yen when sVSP does not occur and when it does, respectively. The greatest savings were observed among nursing staff, with reductions of 37,518.29 yen in cases with sVSP and 33,230.49 yen in cases without. Although the cost reduction associated with clazosentan appeared greater in patients without vasospasm than in those who developed vasospasm, this finding reflects differences in downstream resource utilization rather than a paradoxical effect. In non-vasospasm cases, clazosentan use was associated with fewer rescue interventions and reduced time in high-acuity nursing care, leading to greater cost savings. Conversely, once vasospasm occurred, both groups required similar levels of intensive management, and the incremental drug cost of clazosentan was only partially offset by the reduction in subsequent treatment costs. As a result, the overall cost difference between the two groups was smaller in vasospasm cases. In contrast, the savings for physicians were comparatively modest, particularly when sVSP occurred, resulting in only a 2% reduction in labour costs. However, among patients without sVSP, physician labour costs were reduced by approximately 14% for coiling and 9% for clipping. Clazosentan had a negligible impact on the labour costs of radiologists.

This study employed TDABC method to estimate the human resource costs associated with SAH treatment, comparing the costs of clazosentan with those of conventional therapies. Our findings indicate that clazosentan significantly reduced nursing-related costs by approximately 33%, underscoring its potential to alleviate the burden on nursing staff in the management of SAH. Although physicians play a central role in treatment, clazosentan also led to a reduction of up to 14% in physician labour costs. By lowering the incidence of sVSP, clazosentan reduces the need for additional medical and nursing interventions, ultimately decreasing overall human resource expenditures. These quantified reductions offer a clear demonstration of clazosentan’s economic advantages, supporting its broader clinical adoption. In addition to improving clinical outcomes, clazosentan reduces the financial burden on healthcare systems, presenting a sustainable and cost-effective treatment option for SAH. These findings align with those reported by the National Institute of Public Health, which estimated the incremental cost-effectiveness ratio of clazosentan at 2,886,110 yen per quality-adjusted life year, based on high-quality randomised controlled trials involving Japanese patients [6]. Collectively, the findings affirm the economic value of clazosentan.

Furthermore, the observed reduction in human resource costs—particularly among physicians—suggests a potential to alleviate excessive working hours among healthcare professionals. In Japan, extended working hours in hospitals are a well-documented concern. According to a 2019 survey by the Ministry of Health, Labour and Welfare, 64% of male and 28% of female full-time hospital physicians reported working over 60 h per week. Notably, full-time neurosurgeons worked an average of 61 h and 52 min per week, the second longest among 20 surveyed medical departments [18]. From a practical standpoint, the cost savings observed among nursing staff reflect their intensive involvement in acute SAH care, highlighting opportunities for optimising workforce allocation. Although cost reductions among physicians were smaller, they may still contribute meaningfully to alleviating workload pressures in neurosurgery departments—a critical issue in Japan’s healthcare system. Given the high incidence of SAH in neurosurgical practice, the adoption of treatments, such as clazosentan administration, which reduce both medical and nursing interventions, may contribute to mitigating long working hours by decreasing overall workload. Thus, the present findings underscore not only the economic benefits of clazosentan, but also its potential to improve working conditions for healthcare providers.

TDABC has gained traction in healthcare for its ability to streamline measurement of time and resource utilisation. Unlike traditional costing methods, TDABC facilitates more accurate estimation of labour costs by recording the actual time spent on each activity in the treatment process. For instance, prior studies applying TDABC to the management of heart failure have demonstrated improvements in resource allocation and treatment efficiency [7]. Similarly, a study estimating the costs of joint replacement surgery using TDABC reported more precise cost predictions and enhanced decision-making regarding resource allocation [19]. In the present study, TDABC enabled accurate assessment of time savings and labour cost reductions associated with clazosentan, further supporting its economic value in clinical settings.

A limitation of this study is that process mapping was based on survey data from a single centre, which may limit the generalisability of the findings. While the results clearly highlight the cost-saving potential of clazosentan within one institution, they may not reflect cost structures or clinical workflows in other hospitals or regions of Japan. Moreover, although clazosentan does not directly eliminate the need for neurological monitoring, its preventive effect on vasospasm may lead clinicians and nurses to reduce the frequency of neuro checks in patients considered to be at lower risk. Our simulation incorporated this potential behavioral change as a plausible reflection of real-world practice patterns; however, this assumption may not fully capture variations in clinical decision-making across different institutions. Future research should aim to validate these findings through multicentre studies encompassing a broader range of clinical settings. In addition, although the present cost-effectiveness analysis was based on the results of the Japanese phase 3 trials [4], it should be noted that the recently published REACT study [20] reported less favourable outcomes for clazosentan. The discrepancy between these studies may be attributed to differences in study populations (multinational vs. Japanese), dosing regimens (15 mg/hour vs. 10 mg/hour), and the concomitant use of calcium channel blockers such as nimodipine. Nevertheless, since cost-effectiveness evaluations should be performed within each country’s clinical and economic context, our analysis was appropriately conducted using data derived from Japanese patients.

## Conclusion

Clazosentan use is associated with significantly reduced human resource costs, particularly those associated with nursing. This indicates that, in addition to its clinical efficacy in reducing the incidence of sVSP, clazosentan can lower human resource costs in clinical practice. These results provide economic evidence supporting the potential for more widespread clinical use of clazosentan.
